# Acute Sheehan's Syndrome Presenting with Hyponatremia Followed by a Spontaneous Pregnancy

**DOI:** 10.1155/2022/9181365

**Published:** 2022-11-25

**Authors:** Maria M. Pineyro, Leonardo Diaz, Macarena Guzzetti, Mariana Risso, Jimena Pereda

**Affiliations:** Clinica de Endocrinología y Metabolismo, Hospital de Clínicas, Facultad de Medicina, Universidad de la República, Montevideo, Uruguay

## Abstract

**Background:**

Acute Sheehan's syndrome is rare, as well as hyponatremia as its initial manifestation. In addition, spontaneous pregnancy in patients after Sheehan's syndrome is unusual. To our knowledge, no cases of spontaneous pregnancy after acute Sheehan's syndrome have been reported. We describe a case of Sheehan's syndrome that presented with acute hyponatremia and a spontaneous pregnancy.

**Case:**

A 34-year-old female developed blood loss during delivery, which required a blood transfusion. On day seven postpartum, she presented with headaches, lethargy, and difficulty in breastfeeding. The workup showed hyponatremia (118 mEq/l), secondary hypothyroidism, and low prolactin levels. Magnetic resonance imaging showed pituitary necrosis. She was treated with NaCl, hydrocortisone (cortisol results were not available), and levothyroxine. Laboratory tests six weeks after discharge showed low IGF-1 and 8 AM cortisol and normal FT4, LH, FSH, and PRL levels. She was able to partially breastfeed until 4 months postpartum. Regular menstrual cycles started three months later. She became spontaneously pregnant one year later.

**Conclusion:**

Acute Sheehan's syndrome should be considered in the evaluation of postpartum patients with suggestive symptoms. Physicians should be aware that hyponatremia could be an initial manifestation of Sheehan's syndrome, which requires a high index of suspicion for diagnosis. Spontaneous pregnancy can occur after acute Sheehan's syndrome.

## 1. Introduction

Sheehan's syndrome is a well-known cause of hypopituitarism resulting from postpartum pituitary ischemic necrosis after massive blood loss during delivery [1, 2]. Its frequency has recently declined due to modern obstetric care, more so in developed countries. It is usually diagnosed several years postpartum. Acute presentation is extremely rare and can be life-threatening. Moreover, hyponatremia in the acute form of Sheehan's syndrome is also exceptional. In addition, spontaneous pregnancy in patients with Sheehan's syndrome is very rare [3]. To our knowledge, there are only two reported cases of acute Sheehan's syndrome with a successful pregnancy, one after ovulation induction and the other after in vitro fertilization [1, 4].

We described an extremely rare case of Sheehan's syndrome that presented postpartum with acute severe hyponatremia and a spontaneous pregnancy one year after diagnosis.

## 2. Case Presentation

A 34-year-old female (with 2 previous pregnancies and 1 delivery) with no significant past medical history delivered a healthy, normal-weight male infant at 40 weeks of gestation. At delivery (via the vaginal route), she developed significant blood loss, which required a transfusion of two units of blood. She was discharged 72 hours postpartum. On day seven postpartum, she presented to the emergency department because of headaches, blurred vision, paresthesias, lethargy, and difficulty breastfeeding. On physical examination, she appeared confused, was hypotensive (80/60 mmHg), and was tachycardic.

**Figure 1:**
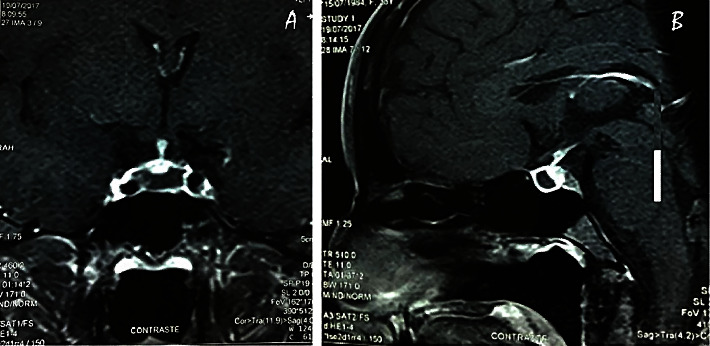
Coronal (a) and sagittal (b) postcontrast MRI at diagnosis. It shows an enlarged pituitary gland with suprasellar extension, peripheral enhancement of the pituitary gland, and irregular and poorly enhanced central portion after gadolinium injection.

Laboratory tests showed hyponatremia of 118 mEq/l (normal range (NR): 138–145) and normocytic normochromic anemia with hemoglobin of 9.7 g/dl (NR: 11.3–14.7). Sheehan's syndrome was suspected based on her clinical presentation with a significant postpartum hemorrhage. Hormone workup revealed secondary hypothyroidism with low free T4 (0.58 ng/dl, NR 0.89–1.76) and normal TSH (0.35 *μ*IU/ml, NR 0.35–5.5) and relatively low prolactin levels (PRL) of 7.12 ng/ml for the early postpartum period (NR for adult women 1.9–25; for women in the third trimester of pregnancy, NR is 84–232). Pituitary MRI showed an enlarged pituitary gland with suprasellar extension, with peripheral enhancement of the pituitary gland, and an irregular and poorly enhanced central portion after gadolinium injection (Figure 1).

She was treated with NaCl replacement to increase her Na levels by 8 mEq/day. Serum 8 AM cortisol was drawn, and as the results were not readily available, treatment with hydrocortisone was initiated with the suspicion of secondary adrenal insufficiency. In addition, levothyroxine treatment was started. Her sodium levels returned to normal, and she was discharged on hormonal replacement therapy (levothyroxine 100 mcg/day, hydrocortisone 15 mg in two divided doses: 10 mg at 8 a.m. and 5 mg at 16: 00 pm).

Laboratory tests six weeks after discharge of the diagnosis of Sheehan's syndrome showed low IGF-1, normal free T4 and LH and FSH levels, PRL levels of 10.2 ng/ml, and serum 8 AM cortisol 24 hours after the last dose of hydrocortisone of 1.72 *μ*g/dl (she did not take the evening dose and took the morning dose after blood was drawn). Cortisol levels at the time of hospitalization before hydrocortisone treatment were 36.2 *μ*g/dl.  Table 1 shows a summary of her hormonal workup. She was able to breastfeed partially until 4 months.

**Figure 2:**
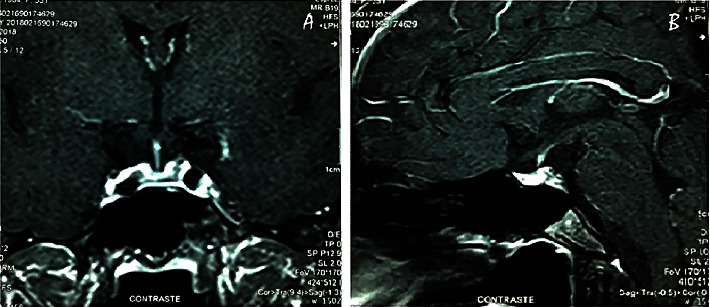
Coronal (a) and sagittal (b) postcontrast MRI 7 months after diagnosis showed a smaller pituitary gland with peripheral enhancement after gadolinium injection.

Regular menstrual cycles started three months after delivery. A repeat MRI performed seven months later showed a smaller pituitary gland with peripheral enhancement after gadolinium injection (Figure 2).

Doses of levothyroxine were gradually decreased with normal FT4 levels when she became spontaneously pregnant one year after the previous delivery. The lowest dose of L-thyroxin on which she became pregnant was 50 mcg 2 days a week and 75 mcg 5 days a week. The dose was increased during pregnancy to 75 mcg per day. The cortisol level was rechecked before pregnancy 24 hours after the last dose of hydrocortisone and was still low (2.54 *μ*g/dl).

She had an uneventful pregnancy on levothyroxine 75 mcg/day and hydrocortisone 15 mg/day in two divided doses. She delivered a healthy, normal-weight female at 39 weeks of gestation.

## 3. Discussion

A diagnosis of Sheehan's syndrome is rarely made in the acute postpartum state. Sheehan's syndrome's true incidence is unknown, and it has been reported to occur in 1%–3.1% of women who lose 1–2 liters of blood associated with hypotension in developed countries [1, 2, 5]. However, prevalence has been reported to be higher in undeveloped countries (3.1%–27.6%) [6–8].

Sheehan's syndrome is generally diagnosed several years postpartum with chronic manifestations such as failure to lactate, amenorrhea, symptoms of hypothyroidism, and adrenal insufficiency. The time between postpartum hemorrhage and symptom onset can be several years. The diagnostic delay is variable. An average delay of 20 ± 8.3 years with a range of 2–36 years was reported in 124 patients with Sheehan's [9]. Ramiandrasoa et al. reported a mean diagnostic delay of 9 ± 9.7 years in 39 women diagnosed with Sheehan's syndrome [10]. Also, Kristjansdottir et al. reported a diagnostic delay from 1 month to 20 years in 8 patients [11].

Acute Sheehan's syndrome is rare, with only 27 cases reported in the literature [1, 3, 12–35] (Table 2).

The first symptoms were reported between 6 hours and 20 days postpartum. Most occur after a massive postpartum hemorrhage and moderate to severe anemia. In one case, severe hypotension was reported at the beginning of the epidural anesthesia without postpartum hemorrhage, followed by panhypopituitarism and diabetes insipidus [15]. In 16 cases, the main presentation was hyponatremia, with a few also presenting with diabetes insipidus, hypothyroidism, panhypopituitarism, and headache. Acute Sheehan's syndrome usually presents with hypotension, tachycardia, failure to lactate, hypoglycemia, fatigue, lethargy, nausea, and vomiting. In the acute phase, hypopituitarism can include ACTH deficiency, which can be fatal if not treated [7].

Hyponatremia is an initial symptom of acute Sheehan's syndrome in approximately 60% of the reported cases. In addition, it is a common electrolyte disturbance in the chronic presentation (33% to 69%) [36–38]. Acute hyponatremia can be life-threatening because it can cause cerebral edema. There are several proposed pathophysiological mechanisms for the development of hyponatremia in this syndrome. It may be due to diminished free water clearance caused by hypothyroidism and adrenal insufficiency [2, 39]. Moreover, a glucocorticoid deficit may enhance the inappropriate secretion of vasopressin. The hypersecretion of vasopressin could be due to the reduction in cardiac output and systemic blood pressure induced by the absence of cortisol. In addition, cortisol deficiency results in increased hypothalamic secretion of corticotropin-releasing hormone (CRH), a vasopressin secretagogue [39, 40]. Furthermore, the negative feedback of cortisol on CRH and ACTH is removed with adrenal insufficiency [41]. Our case presented with secondary hypothyroidism, which may also produce hyponatremia due to reduced free-water clearance.

Cortisol levels were initially normal, but she was pregnant, a condition known to increase CBG levels due to high estrogen levels. In the third trimester of pregnancy, total cortisol levels are increased 2 to 3-fold compared to those of nonpregnant women, which is equivalent to the CBG increase during pregnancy [42]. Cortisol-binding globulin has been reported to normalize between 3 and 6 weeks postpartum [43]. However, others have reported that it remains elevated for 3–6 months [44]. This may be due to its increased half-life. Cortisol levels were low when retested on several occasions. Gonadotropin function was not affected or restored.

Recovery of hormonal function has been reported in the first postpartum year in 2 cases of acute Sheehan's syndrome [13, 22]. In one case, although panhypopituitarism persisted, diabetes insipidus resolved within two years of diagnosis [14]. In our case, while prolactin levels were initially relatively low for the early postpartum period, she was able to partially breastfeed. The latter may be due to a partial lactotroph deficiency or recovered function. This patient had partial hypopituitarism because the gonadotrophic axis was not affected or restored.

The preservation of some hormonal axes could be explained because pituitary necrosis is frequently incomplete, with selective loss of hormone secretion [25]. There can be remnant intact pituitary tissue that continues to receive blood supply from alternative arteries of the neurohypophysis if there is a stop in flow in the superior artery and continues to produce hormones, leading to a selective loss of hormone secretion.

Sheehan's syndrome early MRI findings include a nonhemorrhagic enlargement of the pituitary gland with a thin rim of peripheral enhancement after gadolinium, an intrasellar mass with suprasellar extension, and a lack of enhancement of the pituitary gland [13, 15, 19, 21, 24, 25, 29, 30]. However, in some cases, normal scans were reported in the acute period [1, 22, 23, 26, 27, 33, 34]. After subsequent involution with pituitary shrinkage, atrophic glands and an empty sella are reported in late scans [1, 7, 13, 19, 23–25, 29]. The pituitary gland during pregnancy increases in size and can reach a maximum size of 10 mm during the last trimester and 12 mm in the immediate postpartum period, with an upward symmetric convexity. There is an increase in the T1 signal intensity of the pituitary gland, more so during the third trimester. After the first week postpartum, the pituitary gland returns to its normal size [45].

Sheehan's syndrome pathogenesis is not yet fully understood, with the principal proposed factor being an ischemia of the enlarged anterior pituitary gland during severe peripartum hemorrhage. Due to lactotroph hyperplasia, there is a physiological enlargement of the pituitary gland during pregnancy, reaching 120–136% of its original size [46]. This pituitary hyperplasia is not accompanied by an increase in vascular supply. There is an interruption of arterial blood flow to the gland that may result from arterial spasm due to hypotension resulting from postpartum hemorrhage. In addition, there may be compression of the superior hypophyseal artery because of pituitary enlargement. Moreover, due to hypercoagulation, thrombosis in the pituitary arteries might contribute [7].

Spontaneous pregnancy rarely occurs in patients with Sheehan's syndrome because most patients have hypogonadotropic hypogonadism [47]. Hypogonadotropic hypogonadism has been reported in 67%–100% of patients with Sheehan's syndrome [48, 49]. To our knowledge, no cases of spontaneous pregnancy after acute Sheehan's syndrome have been reported. There are only two reported cases of acute Sheehan's syndrome with a successful pregnancy. One case had ovulation induction, the other in vitro fertilization [1, 4]. In our case, the gonadotropin axis was functional, so pregnancy was a possibility. In a recent review by Zhan et al., 27 patients with Sheehan's syndrome and 32 pregnancies were reported in the literature [4]. Of these pregnancies, 19 (59.4%) were conceived spontaneously, whereas 11 (34.4%) were induced by ovulation. One reported case was due to egg donation [50]. The case reported by Zhan et al. achieved a successful pregnancy by in vitro fertilization and embryo transfer [4].

In this case, we do not believe a lymphocytic hypophysitis complicated with postpartum hemorrhage can be a differential diagnosis, as she did not have symptoms of hypopituitarism and/or of mass lesions prior to the obstetric hemorrhage [51]. In addition, pituitary MRI did not show an enlarged triangular- or dumbbell-shaped gland with a thickened stalk, nor did it enhance homogenously after contrast [52].

In addition, we believe the pituitary apoplexy of an asymptomatic, nonfunctioning pituitary adenoma is not a differential diagnosis either. It presents as sudden and severe headache in more than 80% of cases, with variable degrees of visual-field impairment and ocular palsy in more than 50% of cases (III cranial nerve, most often). Moreover, there were no predisposing risk factors for pituitary apoplexy such as hypertension, coagulopathy, surgery, dynamic testing, or use of dopamine agonists. In addition, an MRI, 7 months later, did not show a residual pituitary adenoma [53]. Furthermore, we do not believe that Rathke's cleft cyst can be a diagnosis either. They are cystic remnants of the craniopharyngeal duct, usually asymptomatic, but they can present with headache, endocrine dysfunction, diabetes insipidus, and visual impairment. In addition, MRI shows a midline cyst usually with T1-hyperintense signal due to proteinaceous mucinous cyst contents [54].

During pregnancy, appropriate hormonal replacement in patients with hypopituitarism is required. Hydrocortisone is the preferred glucocorticoid replacement during pregnancy, as it does not cross the placenta due to its degradation by the 11*β*-hydroxysteroid dehydrogenase 2 enzyme [55]. Higher doses of hydrocortisone may be required during the third trimester.

## 4. Conclusion

Acute presentation of Sheehan's syndrome should be considered in the evaluation of postpartum patients with suggestive symptoms such as hypotension, tachycardia, failure to lactate, hypoglycemia, fatigue, lethargy, nausea, and vomiting. Physicians should be aware that hyponatremia might be an initial manifestation of this entity, which requires a high index of suspicion for diagnosis. Spontaneous pregnancy after acute Sheehan's syndrome is exceedingly rare but can happen in women with preserved gonadal function.

## Figures and Tables

**Table 1:** Hormonal workup.

Date	FT4 (0.89–1.76 mU/ml)	Cortisol (5–25 *μ*g/dl)	LH (2.4–12.6 IU/L)	FSH (2.11–11.3 IU/L)	Prolactin (0.15–25 ng/ml)	Na (135–145 mEq/l)	K (3.5–5.5 mEq/l))
**07/18/17**	0.58	36.2			7.12	118	4.0
**07/28/17**						138	4.1
**08/05/17**						137	
**09/02/17**		1.72	4.11	7.65	10	143	4.0
**05/08/18**							
**07/04/18**	1.05						

Delivery date: 07/11/2017.

**Table 2:** Reported cases of acute Sheehan's syndrome.

Authors	Age (y)	Time of diagnosis related to delivery	Symptoms at presentation	Hypo natremia	Hormone deficit at diagnosis	Hormone recovery	CT scan at diagnosis	CT scan at F/U	Pituitary MRI at diagnosis	Pituitary MRI at follow-up
Putterman et al. [3]	27	7 days	Paresthesia, headache, weakness, somnolence	Yes	LH/FSH, TSH, ACTH	No	Pituitary infarction, increased CSF surrounding gland	Empty sella (6 months postpartum)		
Zuker [12]	20	14 hours	Hypoglycemia	No	ACTH	No	No enhancing pituitary (ischemia)	Small, well enhancing pituitary (3 months after postpartum)		
Lavalle et al. [13]	30	6 hours	Seizures	Yes	TSH, ACTH	TSH, ACTH	Normal	Peripherally enhancing sellar mass with suprasellar extension (5 days after delivery)	Intrasellar mass with suprasellar extension with peripheral enhancement	Atrophic gland (48 days after delivery)
Kan and Calligerous[14]	32	24 hours	Polydipsia and polyuria	No	FSH/LH, TSH, ACTH, ADH	ADH	Normal pituitary gland			
Dejager et al. [15]	32	3 days	Headache, polydipsia, and polyuria	No	GH, LH/FSH, PRL, ADH	No			Holosellar 11-mm mass, pituitary gland did not enhance with contrast, peripheral rim	Shrinkage of pituitary mass of 4 mm, and dissappearance of bright neurophypophysis spot (30 days after delivery
Boulanger et al [16]	30	10 days	Asthenia, failure to lactate	Yes	LH/FSH, TSH, ACTH, PRL	Not reported				
Kale et al. [17]	23	20 days	Psychosis	No	FSH/LH, TSH, ACTH	Not reported		Empty sella		
Schrager et al. [18]	39	12 days	Nausea, vomiting, fatigue, dizziness, diarrhea	Yes	TSH, ACTH	Not reported				
Lust et al. [19]	32	3 days	Headache, failure to lactate	Yes	TSH, ACTH	No			Enlarged pituitary isointense T1 abutting optic chiasm with peripheral gland enhancement	Empty sella (4 months after delivery)
Wang et al. [20]	32	7 days	Polyuria, renal insufficiency	Yes	LH/FSH, TSH, ACTH	Not reported				
Bunch et al. [21]	23	6 days	Fatigue, diffuse abdominal pain, weakness, hypoglycemia	Yes	TSH, ACTH	Not reported			Enlarged pituitary gland, peripheral enhancement	Normal-sized pituitary gland with heterogen-eous uptake of contrast (1 month after delivery)
Munz et al. [22]	33	6 days	Stupor, headache, nausea, vomiting	Yes	TSH, ACTH, PRL	Yes	Normal		Normal	
Wang et al. [23]	33	19 days	Respiratory distress	No	LH/FSH, TSH, ACTH, PRL	Not reported			Normal	Empty sella (day 32 after delivery)
Kaplun et al. [24]	29	17 days	Fatigue, failure to lactate, fever, postural syncope	Yes	GH, LH/FSH, TSH, ACTH, PRL	No			Nonenhancing minimally hypointense lesion in pituitary gland	Empty sella (6 months after delivery)
Kaplun et al. [24]	21	3 days	Headache, fever	Yes	GH, LH/FSH, TSH, ACTH, PRL	No			Enlarged pituitary gland with suprasellar extension, peripheral enhancement with an irregular and poorly enhanced central portion	Empty sella (11 months after delivery)
Anfuso et al. [25]	35	8 days	Asthenia, headache, abdominal pain	Yes	GH, LH/FSH, TSH, ACTH, PRL	No			Lack of enhancement of pituitary gland	Atrophy of adenohypophysis
Kumar et al. [26]	36	4 days	Polyuria	No	TSH, ACTH, PRL, ADH	No			Normal	Normal
Robalo et al. [27]	45	15 days	Headache, fatigue, polydipsia olyuria	No	GH, LH/FSH, TSH, ACTH, PRL, ADH	No	Discrete enlargement of anterior pituitary lobe, hypointense after contrast		Normal pituitary Ectopic posterior pituitary	
Shoib et al. [28]	31	16–18 days	Psychosis, failure to lactate	No	LH/FSH, TSH, ACTH	Not reported		Empty sella		
Sasaki et al. [29]	37	4–6 days	Failure to lactate	Yes	GH, LH/FSH, TSH, ACTH, PRL	No			Slight swelling of anterior lobe and pituitary stalk. There was no enhancement of central portion but a peripheral rim of enhancement	Empty sella
Hale et al. [30]	31	6 days	Severe headaches, failure to lactate, orthostatic hypotension, nausea and vomiting	Yes	TSH, ACTH, PRL, ADH	No			Infarcted enlarged pituitary with an expanded sella, with peripheral rim enhancement after contrast	NA
Matsuzaki et al. [1]	27	8 days	Seizure	Yes	GH, FSH/LH, TSH, ACTH, PRL	No			No abnormalities	Atrophic pituitary gland
Windpessl et al. [31]	31	8 days	Headache, lethargy, postural light-headedness, failure to lactate	Yes	TSH, ACTH	No			Pituitary hyperintense	Pituitary infarction
Rahim et al. [32]	27	2 days	Severe bifrontal headache and photophobia, failure to lactate	No	No	No	Subtly enlarged pituitary gland with an area of focal hypodensity		Acute pituitary infarction	
Meregildo-Rodriguez [33]	24	4 days	Hypoglycemia, seizures	No	GH, FSH/LH, ACTH, TSH, PRL	No	Normal		Normal	
Rahmani Tzvi-Ran et al. [34]	24	1 day	Headache, fatigue, and failure to lactate	Yes	GH, FSH/LH, TSH, ACTH, PRL, ADH	No			Normal pituitary with circumferential enhancement	
Olmes et al. [35]	28	2 days	Failure to lactate, polyuria.	No	FSH/LH, TSH, ACTH, ADH	No				
